# Influence of Temperature and Humidity on the Efficacy of Spinosad Against Four Stored-Grain Beetle Species

**DOI:** 10.1673/031.008.6001

**Published:** 2008-10-06

**Authors:** Christos G. Athanassiou, Nickolas G. Kavallieratos, Alcebiades E. Yiatilis, Basileios J. Vayias, Constantin S. Mavrotas, Željko Tomanović

**Affiliations:** ^1^ Laboratory of Agricultural Zoology and Entomology, Faculty of Plant Science, Agricultural University of Athens; 75 lera Odos str., 11855, Athens, Attica, Greece; ^2^ Laboratory of Agricultural Entomology, Department of Entomology and Agricultural Zoology, Benaki Phytopathological Institute; 8 Stefanou Delta str., 14561, Kifissia, Attica, Greece; ^3^ Department of Plant Production, Technological Educational Institute of Larissa, 41110, Larissa, Greece; ^4^ DOW Agrosciences Export S.A.S.; Thorikon, 19500, Lavrion, Attica, Greece; ^5^ Institute of Zoology, Faculty of Biology, University of Belgrade; Studentski trg 16, 11000 Belgrade, Serbia

**Keywords:** spinosad, wheat, maize, abiotic conditions, grain protectants, *Rhyzopertha dominica*, *Sitophilus oryzae*, *Tribolium confusum*, *Prostephanus truncatus*

## Abstract

In the present work, we examined the insecticidal effect of spinosad, against adults of the lesser grain borer, *Rhyzopertha dominica* (F.) (Coleoptera: Bostrychidae), the rice weevil, *Sitophilus oryzae* (L.) (Coleoptera: Curculionidae), the confused flour beetle, *Tribolium confusum* Jacquelin du Val (Coleoptera: Tenebrionidae) on wheat and the larger grain borer, *Prostephanus truncatus* (Horn) (Coleoptera: Bostrychidae) on maize. The dose rates used were 0.01, 0.1, 0.5 and 1 ppm. The bioassays were carried out at three temperatures, 20, 25 and 30°C and two relative humidity levels, 55 and 75%. Mortality of *R*. *dominica* and *S*. *oryzae* was high even at 0.01 ppm of spinosad, reaching 100% at 55% relative humidity and 30° after 21 days of exposure. Generally, mortality of *R*. *dominica*, increased with temperature while for *S*. *oryzae* mortality increased with temperature and with the decrease of relative humidity. Moreover, for *S*. *oryzae*, mortality was low at 20°C. In the case of *T*. *confusum*, mortality was low at doses between 0.01 and 0.5 ppm even after 21 days of exposure. At 1 ppm, mortality exceeded 90% only at 30°C and only after 21 days of exposure. Mortality of *P*. *truncatus* was low on maize treated with 0.01 ppm, but increasing the dose to 0.1 ppm resulted in > 87% mortality after 14 days of exposure. In several combinations tested, spinosad efficacy notably varied according to the temperature and humidity regimes. Of the species tested, *R*. *dominica* and *P*. *truncatus* were very susceptible to spinosad, followed by *S*. *oryzae*, while *T*. *confusum* was the least susceptible.

## Introduction

The recent registration of several traditional grain protectants and fumigants for health and environmental reasons made it essential to evaluate alternative, reduced risk, methods for stored-grain pest control. Despite the fact that the use of some of these methods has been progressively increased, such as the use of inert materials, biological control or botanicals, stored-grain protection is still based on the use of chemicals. Several new chemical substances with low mammalian toxicity have been evaluated for this purpose in several parts of the world, aiming to gradually replace the use of conventional insecticides, such as the organophosphorates (OPs). For instance, some pyrethroids have been successfully used as alternatives to OPs (Arthur 1996, 1999; Athanassiou et al. 2004; Hagstrum and Subramanyam 2006). Spinosad, which is based on the metabolites from the actinomycete *Saccharopolyspora spinosa* Mertz and Yao (Bacteria: Actinobacteridae), appears to be one of the most promising new grain protectants (Thompson et al. 1997). Spinosad has low mammalian toxicity and acts on the insects' nervous system, by ingestion or contact (Sparks et al. 2001; Subramanyam 2006). So far, spinosad is registered as a grain protectant in the U.S.A. at the labelled rate of 1 ppm (Subramanyam 2006) and is expected that registration for this purpose will be expanded in other parts of the world.

Previous studies document that the insecticidal efficacy of spinosad is affected by several biotic or abiotic factors, such as the target species, the type of commodity, the exposure interval and the type of surface that spinosad is applied to (Fang et al. 2002a; Subramanyam et al. 2003; Toews and Subramanyam 2003; Toews et al. 2003; Nayak et al. 2005; Daglish and Nayak 2006; Getchell 2006; Subramanyam 2006; Subramanyam et al. 2007). So far, spinosad has proved to be very effective against a wide range of stored-product pests, and can retains its efficacy for a long time after application (Fang et al. 2002b; Fang and Subramanyam 2003; Maier et al. 2006; Daglish and Nayak 2006; Subramanyam, et al. 2007). Maier et al. (2006) found that in stored maize, spinosad remained stable for a two year period. However, there is still inadequate information on the effect of temperature and humidity on the insecticidal effect of spinosad against stored-grain pests. These two parameters are crucial for the performance of the currently used grain protectants, since they determine their efficacy (Noble et al. 1982; Johnson 1990; Braness et al. 1998; Arthur 1996, 1999). For example, pyrethroids are generally less effective at high temperatures, while the reverse is true for OPs (Johnson 1990). Musser and Shelton (2005) found that the increase of temperature decreased the efficacy of spinosad against *Ostrinia nubilalis* (Hübner) (Lepidoptera: Crambidae). In the present work, we examined the efficacy of spinosad against four major stored grain pests, three primary colonisers, the lesser grain borer, *Rhyzopertha dominica* (F.) (Coleoptera: Bostrychidae), the rice weevil, *Sitophilus oryzae* (L.) (Coleoptera: Curculionidae) and the larger grain borer, *Prostephanus truncatus* (Horn) (Coleoptera: Bostrychidae), and one secondary coloniser, the confused flour beetle, *Tribolium confusum* Jacquelin du Val (Coleoptera: Tenebrionidae), at three temperatures, 20, 25 and 30°C and two relative humidity levels, 55 and 75%.

## Materials and Methods

### Test insects

All species used were kept in the laboratory at 26 ±1°C, 65 ± 5% relative humidity and continuous darkness. *R*. *dominica* and *S*. *oryzae*, were reared on whole wheat, while *T*. *confusum* was reared on wheat flour plus 5% brewer's yeast (by weight). *P*. *truncatus* was reared on whole maize. For all species, adults used in the tests were less than 4 weeks old.

### Commodities

Untreated, clean and infestation-free wheat (var. Dion) and maize (var. Dias) were used in the tests. The initial moisture content of the grains, as determined by a moisture meter (Dickey-John multigrain CAC-II, www.dickey-john.com) was approx. 11.1%. Before the beginning of the experiments, the grain was left at ambient conditions (see below) for 7 days, to equilibrate with the relative humidity level.

### Formulation

The spinosad formulation, named NAF-313, contained 11.8% of a.i., as emulsifable concentrate (EC; d = 1.0374 g/ml). This formulation was provided by DOW Agrosciences Export S.A.S. (Lavrion, Attica, Greece).

### Grain treatment

Spinosad was applied as solution (diluted in distilled water) on wheat or maize at four dose rates: 0.01, 0.1, 0.5 and 1 ppm. For each grain and dose, 1 kg lots were prepared. To achieve the desired doses, 30 ml aqueous solutions of spinosad were prepared by adding, to the appropriate quantity of spinosad, distilled water up to 30 ml volume. Spraying was carried out on a tray, on which the 1 kg of grain was spread into a thin layer. For each grain, there was an additional untreated lot that served as control (sprayed with 30ml distilled water only).

### Bioassays

Four samples of 60 g each were taken from each wheat lot, and placed in small cylindrical glass vials (16 cm in diameter, 28 cm in height), which had a hole, 1.5 cm in diameter, covered with organdy for aeration. 30 adult beetles were introduced into each vial. The vials were then placed in incubators, set at 20°C, 55% relative humidity and continuous darkness. Dead adults were counted after 7, 14 and 21 days of exposure in treated, and untreated, grains. The test was repeated three times (3 × 4 vials), using new lots of grains each time. The tests were conducted at three temperatures, 20, 25 and 30°C and two relative humidity levels, 55% and 75%. This procedure was followed for *R*. *dominica*, *S*. *oryzae* and *T*. *confusum* adults. In the case of *P*. *truncatus*, maize was used, since this species is a primary pest of maize, despite the fact that it can be found in other grain commodities as well (Boxall 2002).

### Data analysis

Control mortality was corrected by using Abbott's (1925) formula. Mortality in the control vials for the species tested ranged between 5–10%. For each species, the mortality data were analyzed by the GLM procedure of SAS (SAS Institute 1995), with insect mortality as the response variable and temperature, relative humidity, dose rate and exposure interval as main effects. For the comparison of the means, the Tukey-Kramer HSD test at P < 0.05 was used (Sokal and Rohlf 1995).

## Results

### Mortality of *R*. *dominica*

All main effects, with the exception of relative humidity were significant at the P < 0.0001 level, while from the associated interactions temperature × dose and relative humidity × exposure were not significant (Table 1).

After 7 days of exposure, at 0.01 ppm, mortality of the exposed *R*. *dominica* adults ranged between 21 and 56% (Table 2). Generally, with the exception of 20°C, no significant differences were noted between the two relative humidity levels tested. Moreover, increasing temperature increased beetle mortality. However, at 75% relative humidity, mortality at 30°C was significantly higher than that at 25°C but not than that at 20°C. At the 14-day interval, mortality was > 83% at 30°C, but significant differences among temperatures were noted only at 55% relative humidity. Finally, 7 days later, at 55% relative humidity, all adults were dead at 30°C but only 68% had died at 20°C, while at 75% relative humidity, mortality was > 90% at all temperatures tested.

**Table I.**
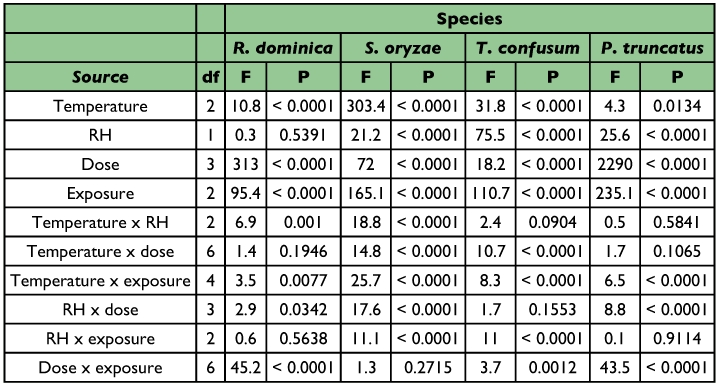
ANOVA parameters for main effects and interactions for the species tested (total df = 647)

On wheat treated with 0.1 ppm of spinosad, after 7 days of exposure, no significant differences were noted among relative humidity levels. At 55% relative humidity, significantly fewer adults were dead at 20°C than at 30°C, where mortality reached 98%. At 75% relative humidity, the increase in temperature significantly increased mortality, while all adults were dead at 30'C Seven days later for all temperature - relative humidity combinations, all adults were dead, with the exception of 25°C at 55% relative humidity, where mortality was 99.6%.

A further increase of spinosad dose to 0.5 ppm, after 7 days of exposure, gave high mortality levels, ranging between 90 and 100%. Also, as above, no significant differences were noted between the two relative humidity levels. At the 14-day exposure interval, all adults were dead, with the exception of 20°C 75%, where mortality was 99%.

Finally, on wheat treated with 1 ppm, after 7 days of exposure, > 95% of the exposed adults were dead, while no differences were noted between relative humidity levels. In all combinations, mortality was 100% at the 14-day exposure.

### Mortality of *S*. *oryzoe*

All main effects and all associated interactions, with the exception of dose × exposure, were significant (Table 1).

At 0.01 ppm of spinosad, after 7 days of exposure, mortality varied remarkably from 15 to 94%, while, at both relative humidity levels, mortality increased with the increase of temperature (Table 3). Thus, at 30°C, 87–94% of the exposed weevils died, while at 20°C, mortality did not exceed 24%. Similar trends were also noted 7 days later. At 55% relative humidity, all weevils were dead at 30°C, but only 60% at 20°C while the respective figures for 75% relative humidity were 34 and 90%. Generally, mortality was lower at 75% than at 55% relative humidity. After 21 days of exposure, mortality increased very little at 20°C, while at the other two temperatures, mortality significantly decreased with the increase of relative humidity.

**Table 2.**
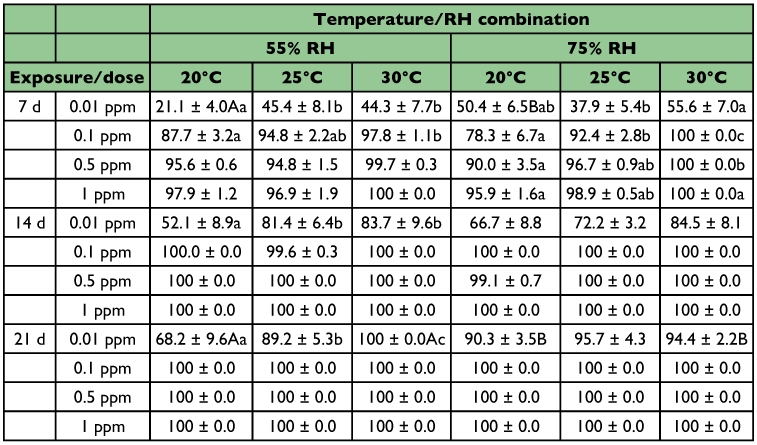
Mean (%) mortality (± SE) of *R*. *dominica* adults exposed on wheat treated with four dose rates of spinosad at three temperatures and two RH levels (within each row and RH, means among temperatures followed by the same lower case letter are not significantly different, within each row and temperature, means between RH levels followed by the same upper case letter are not significantly different; where no letters exist, no significant differences were noted; HSD test at 0.05)

**Table 3.**
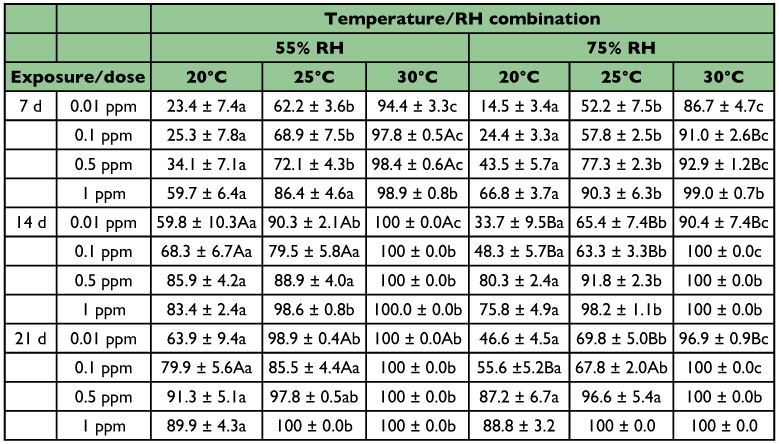
Mean (%) mortality (± SE) of *S*. *oryzoe* adults exposed on wheat treated with four dose rates of spinosad at three temperatures and two RH levels (within each row and RH, means among temperatures followed by the same lower case letter are not significantly different, within each row and temperature, means between RH levels followed by the same upper case letter are not significantly different; where no letters exist, no significant differences were noted; HSD test at 0.05)

**Table 4.**
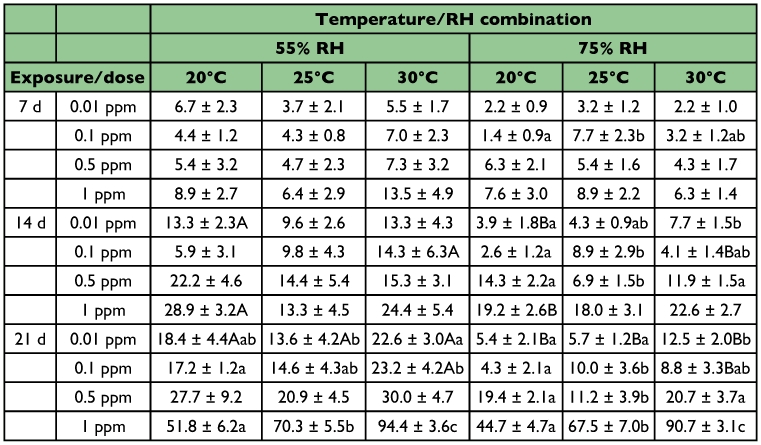
Mean (%) mortality (± SE) of *T*. *confusum* adults exposed on wheat treated with four dose rates of spinosad at three temperatures and two RH levels (within each row and RH, means among temperatures followed by the same lower case letter are not significantly different, within each row and temperature, means between RH levels followed by the same upper case letter are not significantly different; where no letters exist, no significant differences were noted; HSD test at 0.05)

On wheat treated with 0.1 ppm of spinosad, after 7 days of exposure, mortality increased very little (by 1.9 to 9.9%) in comparison with 0.01 ppm, while as above, the increase of temperature increased mortality (Table 3). At 25°C, mortality was 69 and 58% at 55 and 75% relative humidity, respectively. The effect of relative humidity was significant only at 30°C. At the 14-day exposure interval, all weevils were dead at 30°C, while at the other two temperatures, mortality was higher at 55 than at 75% relative humidity. Finally, after 21 days of exposure, similar trends were also recorded, while mortality at 25°C did not exceed 86%.

At 0.5 ppm, after 7 days of exposure, mortality was > 92% at 30°C, but it did not exceed 44% at 20°C Table 3). Also significant differences between the two relative humidity levels were noted only at 30°C. After 14 days of exposure, the increase of temperature increased mortality, but > 80% of the exposed weevils were dead even at 20°C. Similarly, 7 days later, > 87% of the exposed weevils were dead, but mortality did not reach 100% at 25°C.

At the highest spinosad dose, after 7 days of exposure, mortality was > 86% at ≥ 25°C, but it did not exceed 67% at 20°C (Table 3). Mortality was > 75% after 14 days of exposure, while one week later, all weevils were dead at 25 and 30°C. It should be noted that, at this dose rate, mortality did not differ significantly between the two relative humidity levels.

### Mortality of *T*. *confusum*

All main effects were significant at the P < 0.0001 level, while of the associated interactions, temperature × relative humidity and relative humidity × dose were not significant (Table 1).

At 0.01 ppm, at the 7-day exposure interval, mortality was extremely low and did not exceed 7% (Table 4). These low levels of efficacy continued also at the other two exposure intervals. After 21 days of exposure, mortality ranged between 5 and 22%. At this interval, mortality was higher at 55% than at 75% relative humidity. Furthermore, despite the increase of the dose rate to 0.1 ppm or to 0.5 ppm, mortality remained at low levels, even after the 21 days of exposure.

At 1 ppm, after 7 days of exposure, mortality was low (<14%), but two weeks later > 90% of the exposed adults were dead at 30°C (Table 4). At this exposure, the increase of temperature increased mortality, while no significant differences were noted between the two relative humidity levels tested.

### Mortality of *P*. *truncatus*

All main effects were significant while of the associated interactions, temperature × relative humidity, temperature × dose and relative humidity × exposure were not significant (Table 1).

On maize treated with 0.01 ppm of spinosad, mortality of *P*. *truncatus* was low (< 21%), while significant differences were noted among temperatures and relative humidity (Table 5). One week later, no significant differences were noted in mortality levels among the three temperatures at 55% relative humidity, while at 75% relative humidity, mortality increased with temperature. At the 21 days of exposure, as previously, no significant differences were noted among temperatures at 55% relative humidity, while mortality reached 49% at 20°C.

**Table 5.**
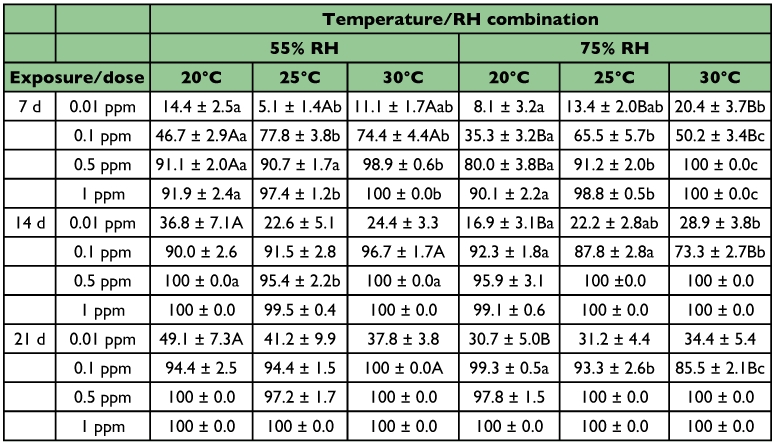
Mean (%) mortality (± SE) of *P*. *truncatus* adults exposed on maize treated with four dose rates of spinosad at three temperatures and two RH levels (within each row and RH, means among temperatures followed by the same lower case letter are not significantly different, within each row and temperature, means between RH levels followed by the same upper case letter are not significantly different; where no letters exist, no significant differences were noted; HSD test at 0.05)

On maize treated with 0.1 ppm, after 7 days of exposure, at 55% relative humidity, significantly more adults were dead at 25 and 30°C, where mortality was > 74%, as compared with 20°C, where mortality was 47% (Table 5). In contrast, at 75% relative humidity, this trend was partially reversed, and mortality at 25°C was significantly higher than at the other two temperatures. Also mortality was higher at 55% than at 75% relative humidity. At the 14-day exposure interval, at 55% relative humidity, mortality was ≥ 90%, while no significant differences were noted among temperature levels. In contrast, at 75% relative humidity, mortality at 30°C was significantly lower than at the other two temperatures. Similar trends were also recorded in the case of the 21-day exposure interval. Hence, mortality at 75% relative humidity was > 93% at ≤ 25°C, but only 86% at 30°C On the other hand, at 55% relative humidity, mortality was high (> 94%) and reached 100% at 30°C.

On maize treated with 0.5 ppm, after 7 days of exposure, mortality increased with temperature at both relative humidity levels, and with one exception (20°C 55% relative humidity) it ranged between 80 and 100% (Table 5). Similarly, after 14 days of exposure, mortality ranged between 95 and 100%. Finally, after 21 days of exposure, all adults were dead, with the exception of 25°C, 55% relative humidity and 20°C, 75% relative humidity, where mortality was 97.2 and 97.8% respectively. The increase of dose to 1 ppm increased further the adult mortality levels that reached 100% after 7 days of exposure at 30°C. After 21 days of exposure, a complete (100%) mortality was noted in all temperature/relative humidity combinations examined.

## Discussion

The results of the present study indicate that there was a significant impact of temperature and relative humidity on spinosad efficacy against all species tested. The influence of these two parameters is complex since it varied according to the dose rate and the exposure interval. Of the four species tested, *R*. *dominica* was the most susceptible to spinosad, which is in accordance with the results published by other researchers (Fang et al. 2002a; Subramanyam et al. 2003; Toews and Subramanyam 2003; Nayak et al. 2005; Daglish and Nayak 2006). For instance, Fang and Subramanyam (2003) found that 0.1 ppm of spinosad on wheat caused 100% mortality of *R*. *dominica* adults and notably suppressed progeny production. Nayak et al. (2005) tested several *R*. *dominica* strains with various susceptibility levels to some of the most commonly used grain protectants and found that 0.1 ppm of spinosad provided 93 – 99% adult mortality after 7 days of exposure, regardless of the resistance status to other pesticides of the beetles tested. These findings are particularly important, since this species has developed a considerable level of resistance to many currently used grain protectants (Champ and Dyte 1976; Zettler and Cuperus 1990; Collins et al. 1993). The high susceptibility of *R*. *dominica* to spinosad led us to investigate if this species is susceptible at doses lower than 0.1 ppm. The results suggest that high levels of mortality could be obtained at 0.01 ppm, although mortality was delayed in comparison with the 0.1 ppm dose, which confirms the high susceptibility of this species to spinosad.

In general, adult mortality of *R*. *dominica* increased with temperature. However, temperature played an important role in the case of low dose rates or short exposure intervals. At high dose rates, or long exposures, the temperature effect disappeared, probably due to the high mortality levels. In a previous study, Fang and Subramanyam (2003) noted that *R*. *dominica* mortality on spinosad treated wheat was not affected by temperature. In that study, the authors used spinosad at 0.1 and 1 ppm and measured mortality after 14 days of exposure when they found it to be 100%. Our results are in agreement with these findings, since no significant differences were noted among temperatures for doses ≥ 0.1 ppm, at exposures ≥ 14 days. Hence, we can conclude that, despite the fact that in the present study a temperature effect of spinosad was observed against *R*. *dominica*, this effect was rather weak due to the high effectiveness of spinosad and may appear only at low dose rates (e.g. < 0.1 ppm) or short exposures (e.g. 7 days). The same holds for relative humidity; significant differences were noted only at 0.01 pm of spinosad, and only at certain exposure intervals, while at dose rates 0.1 ppm or higher, spinosad was equally effective against *R*. *dominica*. Fang and Subramanyam (2003) also found that spinosad efficacy was not affected by wheat moisture content. Similarly, Daglish and Nayak (2006) found that, at 30°C, relative humidity had no effect on *R*. *dominica* mortality and progeny production, over a period of 9 months after the spinosad application. Consequently, we can conclude that, of the four species tested here, *R*. *dominica* was least affected by temperature and relative humidity.

Like *R*. *dominica*, *S*. *oryzae* is also resistant to many of the currently used grain protectants (Champ and Dyte 1976; Collins et al. 1993). Furthermore, the females of this species oviposit in the internal part of the kernel, which makes it difficult its control with Insect Growth Regulators, which normally control other stored grain insect species (Daglish and Wallbank 2005). Hence, the use of an alternative grain protectant is particularly important in the case of this species. Spinosad has been proved effective against this species (Fang et al. 2002a; Subramanyam et al. 2003; Toews et al. 2003; Nayak et al. 2005; Getchell 2006). Nevertheless, the effect of temperature and relative humidity on the efficacy of spinosad against this species has not been examined in detail so far. According to the results of the present study, spinosad efficacy against *S*. *oryzae* is notably affected by temperature and, to a lesser extent, by relative humidity. In fact, the influence of temperature was greater against *S*. *oryzae* than against the other three species tested. The increase of temperature from 20 to 30°C remarkably increased weevil mortality, even at the lowest dose rate used. At 20°^0^C, mortality was low even at low spinosad doses. On the other hand, at 30°C, spinosad was extremely effective; for instance, at 55% relative humidity, on wheat treated with 0.01 ppm, 94% of the exposed individuals died after only 7 days of exposure, while the respective figure at 20°C was only 24%. As *S*. *oryzae* adults are much more agile than *R*. *dominica*, the increase of temperature increases mobility. Hence, since spinosad acts also as a contact insecticide, we assume that at 30°C the contact of weevils with the toxic agent increased, resulting in increased mortality. Moreover, 30°C is close to the developmental optima of this species (Aitken 1975), hence increased body metabolic activity at warm conditions may have resulted in the increased susceptibility to spinosad. From a practical point of view, 0.5 and 1 ppm of spinosad gave similar mortality levels. Consequently, the benefit of an increase of the spinosad dose to 1 ppm was low, and 0.5 ppm can satisfactorily control *S*. *oryzae*, provided that the prevailing temperatures are ≥ 25°C. Also, in some of the combinations tested, the increase of relative humidity level decreased spinosad efficacy, especially at low dose rates (0.01 and 0.1 ppm). Generally, *S*. *oryzae* develop better at relatively high relative humidity levels, close to 75% (Aitken 1975) and at low relative humidity levels, insects may be more stressed, which may result in increased mortality after exposure to spinosad. Nevertheless, the results obtained in the present study are representative only for the specific strain and commodity tested, and thus, generalizations should be avoided. Fang et al. (2002a), after 7 days of exposure, found 100% mortality on durum wheat treated with 1 ppm of spinosad, but only 69–76% to other classes of wheat. Also, Nayak et al. (2005) reported that the efficacy of spinosad varied remarkably among different *S*. *oryzae* strains.

In contrast with *R*. *dominica* and *S*. *oryzae*, very little data exist on the efficacy of spinosad against *T*. *confusum*. For the red flour beetle, *Tribolium castaneum* (Herbst) (Coleoptera: Tenebrionidae) the available data indicate that this species is among the least susceptible storedgrain insect species to spinosad. For instance, Toews and Subramanyam (2003) reported that *T*. *castaneum* was by far more tolerant to spinosad than *R*. *dominica* and *S*. *oryzae*. Similar results have also been reported by other researchers as well (Fang et al. 2002a; Huang et al. 2004; Nayak et al. 2005; Getchell 2006). Our results agree that *T*. *confusum* is not very susceptible to spinosad and survival can be high even at high dose rates and increased exposure intervals. High mortality (> 90%) was observed only at 30°C and only on wheat treated with 1 ppm of spinosad after 21 days of exposure. Toews et al. (2003), tested the susceptibility of eight stored-product beetle species to surfaces treated with spinosad, classified *T*. *castaneum* and *T*. *confusum* as the least susceptible species. In fact, in that study, the authors reported that *T*. *castaneum* was less susceptible than *T*. *confusum*. Generally *T*. *confusum* was found to be less affected by these two variables than *S*. *oryzae*, although in some of the combinations tested, the increase of temperature and the decrease of relative humidity increased susceptibility to spinosad.

*P*. *truncatus* is a species of major importance in Africa, where it causes serious damage each year, particularly to maize (Boxall 2002). One of the reasons that causes its control measures to fail is the remarkable resistance of this species to some of the currently used insecticides (Golob 2002), and also to some alternatives, such as diatomaceous earth (Athanassiou et al. 2007). Hence, the evaluation of alternative control agents against this species is essential. Very few data are also available on the susceptibility of *P*. *truncatus* to spinosad. Mutambuki et al. (2003) tested spinosad against *P*. *truncatus* in semi-field tests in Kenya. The authors used a spinosad dust formulation at rates of 0.35, 0.70 and 1.44 ppm on maize, which was very effective against this species for a 6-month period; in fact, at the end of this interval spinosad was more effective than 10.5 ppm of “Actellic super dust”, which is a pirimiphos-methyl/permethrin combination. In the present work, 0.01 ppm of spinosad was not effective against *P*. *truncatus*, even at long (21 day) exposure intervals. Nevertheless, doses ≥ 0.1 ppm caused very high mortality, which reached, in some cases, 100% even after 7 days of exposure. In several combinations tested spinosad efficacy notably varied according to the temperature and relative humidity level. Taking into account the overall data, spinosad was less effective under humid conditions. Although spinosad at 0.1 ppm was effective, mortality at this dose was delayed, while at 0.5 ppm, with one exception, mortality was > 90% even after 7 days of exposure. Moreover, the mortality data indicate that increasing the dose to 1 ppm contributed very little to the overall mortality. Thus, these data indicate that 0.5 ppm of spinosad can be used with success against this species in stored maize. Taking into consideration the current results and the results reported by Mutambuki et al. (2003) *P*. *truncatus* can be classified as among the most susceptible stored-grain insect species to spinosad. Nevertheless, although some general trends can be drawn, direct comparisons with the other three species tested here may be inaccurate, since a different commodity, corn, was used in the case of this species.

In summary, our results indicate that spinosad is an effective tool in stored-product protection, but its effectiveness depends on the target species (Subramanyam 2006). The four species tested here, from the most susceptible to the most tolerant to spinosad, can be ranked as *R*. *dominica* > *P*. *truncatus* > *S*. *oryzae* > *T*. *confusum*. Practically, insect identification is one of the key elements in designing an IPM-based control strategy, since the species itself determines the dose rate that is to be used. These results suggest that *R*. *dominica* can be satisfactorily controlled with 0.1 ppm of spinosad, while 0.5 ppm is needed to control *P*. *truncatus* and *S*. *oryzae*. Doses of 1 ppm or even higher may be required to control *T*. *confusum*. Temperature and relative humidity also seem to play an important role in spinosad efficacy. Generally, although spinosad efficacy was not affected to the same degree among the four species tested, the data suggest that spinosad is more effective at high temperatures and low relative humidity levels.

## References

[bibr01] Abbott WS (1925). A method of computing the effectiveness of an insecticide.. *Journal of Economic Entomology*.

[bibr02] Aitken AD (1975). Insect Travelers, I: Coleoptera.. *Technical Bulletin*.

[bibr03] Arthur FH (1996). Grain protectants: current status and prospects for the future.. *Journal of Stored Products Research*.

[bibr04] Arthur FH (1999). Effect of temperature on residual toxicity of cyfluthrin wettable powder.. *Journal of Economic Entomology*.

[bibr05] Athanassiou CG, Papagregoriou AS, Buchelos CTh (2004). Insecticidal and residual effect of three pyrethroids against *Sitophilus oryzae* (L.) (Coleoptera: Curculionidae) on stored wheat.. *Journal of Stored Products Research*.

[bibr06] Athanassiou CG, Kavallieratos NG, Peteinatos GG, Petrou SE, Boukouvala MC, Tomanovi Ž (2007). Influence of temperature and humidity on insecticidal effect of three diatomaceous earth formulations against the larger grain borer (Coleoptera: Bostrychidae).. *Journal of Economic Entomology*.

[bibr07] Boxall RA (2002). Damage and loss caused by the larger grain borer *Prostephanus truncatus*.. *Integrated Pest Management Reviews*.

[bibr08] Braness GA, Coster DC, Bennett GW (1998). Logistic models describing effects of temperature and humidity on residual effectiveness of chlorpyrifos and cyfluthrin formulations against German cockroaches (Dictyoptera: Blatellidae).. *Journal of Economic Entomology*.

[bibr09] Champ BR, Dyte CE (1976). *Report of the FAO global survey of pesticide susceptibility of stored grain pests*.

[bibr10] Collins PJ, Lambkin TM, Bridgeman BW, Pulvirenti C (1993). Resistance to grain protectant insecticides in coleopterous pests of stored cereals in Queensland, Australia.. *Journal of Economic Entomology*.

[bibr11] Daglish GJ, Wallbank BE (2005). Efficacy of diflubenzuron plus methoprene against *Sitophilus oryzae* and *Rhyzopertha dominica* in stored sorghum.. *Journal of Stored Products Research*.

[bibr12] Daglish GJ, Nayak MK (2006). Long-term persistence and efficacy of spinosad against *Rhyzopertha dominica* (Coleoptera: Bostrychidae) on wheat.. *Pest Management Science*.

[bibr13] Fang L, Subramanyam Bh (2003). Activity of spinosad against adults of *Rhyzopertha dominica* (F.) (Coleoptera: Bostrichidae) is not affected by wheat temperature and moisture.. *Journal of the Kansas Entomological Society*.

[bibr14] Fang L, Subramanyam Bh, Arthur FH (2002a). Effectiveness of spinosad on four classes of wheat against five stored product insects.. *Journal of Economic Entomology*.

[bibr15] Fang L, Subramanyam Bh, Bolder S (2002b). Persistence and efficacy of spinosad residues in farm stored wheat.. *Journal of Economic Entomology*.

[bibr16] Getchell AI (2006). Efficacy of two spinosad formulations on various commodities against stored product insects.*MSc Thesis*.

[bibr17] Golob P (2002). Chemical, physical and cultural control *Prostephanus truncatus*.. *Integrated Pest Management Reviews*.

[bibr18] Hagstrum DW, Subramanyam Bh (2006). *Fundamentals in stored*-*product entomology*.

[bibr19] Huang F, Subramanyam B, Toews MD (2004). Susceptibility of laboratory and field strains of four stored product insect species to spinosad.. *Journal of Economic Entomology*.

[bibr20] Johnson DL (1990). Influence of temperature on toxicity of two pyrethroids to grasshoppers (Orthoptera: Acrididae).. *Journal of Economic Entomology*.

[bibr21] Maier DE, Ileleji KE, Szabela D., Lorini I, Bacaltchuk B, Beckel H, Deckers E, Sundfeld E, dos Santos JP, Biagi JD, Celaro JC, Faroni LRD'A, Bortolini L, de OF, Sartori MR, Elias MC, Guedes RNC, da Fonseca RG, Scussel VM (2006). Efficacy of spinosad for insect management in stored maize.. *Proceedings of the 9th International Working Conference for Stored*-*Product Protection*.

[bibr22] Musser FR, Shelton AM (2005). The influence of post-exposure temperature on the toxicity of insecticides to *Ostrinia nubilalis* (Lepidoptera: Crambidae).. *Pest Management Science*.

[bibr23] Mutambuki K, Ngatia CM, Mbugua JN, Likhayo P, Credland PF, Armitage DM, Bell CH, Cogan PM, Highley E (2003). Evaluation on the efficacy of spinosad dust against major storage pests.. *Proceedings of the 8th International Conference on Stored*-*Product Protection*.

[bibr24] Nayak MK, Daglish GJ, Byrne VS (2005). Effectiveness of spinosad as a grain protectant against resistant beetle and psocid pests of stored grain in Australia.. *Journal of Stored Products Research*.

[bibr25] Noble RM, Hamilton DJ, Osborne WJ (1982). Stability of pyrethroids on wheat in storage.. *Pesticide Science*.

[bibr26] SAS Institute (1995). *SAS user's guide*: *statistics.*.

[bibr27] Sokal RR, Rohlf FJ (1995). *Biometry*.

[bibr28] Sparks TC, Crouse GD, Durst G (2001). Natural products as insecticides: the biology, biochemistry and quantitative structure-activity relationships of spinosyns and spinosoids.. *Pest Management Science*.

[bibr29] Subramanyam Bh, Toews MD, Fang L, Credland PF, Armitage DM, Bell CH, Cogan PM, Highley E (2003). Spinosad: an effective replacement for organophosphate grain protectants.. *Proceedings of the 8th International Conference on Stored*-*Product Protection*.

[bibr30] Subramanyam Bh, Lorini I, Bacaltchuk B, Beckel H, Deckers E, Sundfeld E, dos Santos JP, Biagi JD, Celaro JC, Faroni LRD'A, Bortolini L, de OF, Sartori MR, Elias MC, Guedes RNC, da Fonseca RG, Scussel VM (2006). Performance of spinosad as a stored grain protectant.. *Proceedings of the 9th International Working Conference for Stored*-*Product Protection*.

[bibr31] Subramanyam Bh, Toews MD, Ileleji KE, Maier DE, Thompson GD, Pitts TJ (2007). Evaluation of spinosad as a grain protectant on three Kansas farms.. *Crop Protection*.

[bibr32] Thompson GD, Michel KH, Yao RC, Mynderse JS, Mosburg CT, Worden TV, Chio EH, Sparks TC, Hutchins SH (1997). The discovery of *Saccharopolyspora spinosa* and a new class of insect control products.. *Down to Earth*.

[bibr33] Toews MD, Subramanyam Bh (2003). Contribution of contact toxicity and wheat condition to mortality of stored product insects exposed to spinosad.. *Pest Management Science*.

[bibr34] Toews MD, Subramanyam Bh, Rowan JM (2003). Knockdown and mortality of adults of eight species of stored product beetles exposed to four surfaces treated with spinosad.. *Journal of Economic Entomology*.

[bibr35] Zettler JL, Cuperus GW (1990). Pesticide resistance in *Tribolium castaneum* (Coleoptera: Tenebrionidae) and *Rhyzopertha dominica* (Coleoptera: Bostrichidae) in wheat.. *Journal of Economic Entomology*.

